# Supplementary material for: Seven naturally variant loci serve as genetic modifiers of Lamc2jeb induced non-Herlitz junctional Epidermolysis Bullosa in mice
Source: PLoS One. 2023 Jul 12;18(7):e0288263. doi: 10.1371/journal.pone.0288263 (PMC10337971; doi:10.1371/journal.pone.0288263)
Supplement: Attachment — Submitted filename: Requested rebuttal letter.docx [file pone.0288263.s003.docx]

Requested rebuttal letter

March 28, 2023

To the editor of PLOS One:

This concerns submission PONE-D-23-09055, Seven naturally variant loci serve as genetic modifiers of Lamc2jeb induced non-Herlitz Junctional Epidermolysis Bullosa in mice, which you correctly noted as a manuscript previously submitted to PLOS Genetics as PGENETICS-D-23-00236.

An email from PLOS ONE to me said the following “It is a requirement by the journal that any manuscript resubmitted after a previous round of review is accompanied by a rebuttal letter outlining the response to the original reviewers’ comments, so that the editors can establish whether the original concerns have been addressed. Can you therefore please provide us with a point-by-point response to the comments provided by the original reviewers, outlining the revisions carried out since the original submission.”

The Final Decision email from PLOS Genetics brought up a single point: “The manuscript has been reviewed and discussed internally by members of the editorial board. While there is enthusiasm for both the general question (natural variants as modifier genes) and specific question (pathophysiology of blistering diseases) being addressed, there is also a consensus that additional work to identify one or more of the causal variants and new loci would be necessary for the manuscript to move forward at PLOS Genetics.”

My response is as follows:

A decision of whether to publish QTL findings with suggested variants alone or require them to include firm identification of the causal variant is at the discretion of each journal. We *have* performed publishable work that identifies a causal variant in dystonin, one of the six QTL identified in this manuscript, but both that manuscript and this are lengthy, and we feel it is best to submit them as separate works rather than trying to condense and combine their findings in a single manuscript. Our intent is to submit the ‘dystonin paper’ somewhere once this manuscript has been accepted for publication.

Based on the wording of the PLOS Genetics Final Decision letter, we felt they would not accept this as a reason for them to publish the QTL findings as a stand-alone work. Therefore we opted to submit to PLOS ONE, as a journal which might consider this evidence for six new modifier QTL as worth publishing independent of the follow-up work. The manuscript submitted to PLOS ONE is nearly identical to the one previously submitted to PLOS Genetics, without any changes in response to their comments.

We would be amenable to discussing with PLOS ONE or PLOS Genetics the possibility of publishing the two works concurrently if that would increase your interest in publishing the QTL work.

Let me know if this satisfies your concerns or if you need additional information.

Sincerely,

Thomas J Sproule
