# Supplementary material for: Seven naturally variant loci serve as genetic modifiers of Lamc2jeb induced non-Herlitz junctional Epidermolysis Bullosa in mice
Source: PLoS One. 2023 Jul 12;18(7):e0288263. doi: 10.1371/journal.pone.0288263 (PMC10337971; doi:10.1371/journal.pone.0288263)
Supplement: Attachment — Submitted filename: Response to Reviewers.docx [file pone.0288263.s004.docx]

Response to Reviewers

Thank you for your thoughtful comments concerning our submitted manuscript *Seven naturally variant loci serve as genetic modifiers of Lamc2jeb induced non-Herlitz Junctional Epidermolysis Bullosa in mice.*

Reviewer 1.

General comments:

“It may be helpful to include a summary Table or Figure that details predicted (or known) genotypes for each QTL along with predicted effector gene(s).”

Response: Table 1 has been added to summarize the modifier effects of all QTL and the total effects for each of the four strains used in this study: 129X1, B6, FVB and MRL.

“In the final two sentences of the Discussion section, the authors provide solid rationale for how the data obtained in this study could be applied translationally to improve patient outcomes. It would be beneficial to include this rationale in the Introduction and/or Abstract as well to highlight its importance.”

Response: The overall intent of this manuscript was to communicate the importance of multiple genetic modifiers in determining the severity of EB. Those with the strongest effect were related to structural proteins, while others showing a weaker but significant effects were consistent with regulatory/signaling proteins. We think it would be premature to overstress the therapeutic potential of targeting such proteins at this point.

Specific comments:

“Page 4, Line 75: The abbreviation "LTA" is not previously defined and is not obvious.”

Response: We have amended the text (LTA=Lymphotoxin alpha).

“Page 9, Lines 166-167: It would be helpful to clarify that the short congenic strain was generated as part of the current study.”

Response: In page 9 we have clarified that the B6-*Lamc2^jeb/jeb^* short congenic was generated as part of this study.

“Page 11, Lines 192-194: Inclusion of body weight as a covariant may be appropriate, but it is not immediately clear how this variable would influence tail tension test results or why it was considered as a potential covariant, aside from the substantial variation observed.”

Response: JEB, like other forms of EB, manifests not only in the skin but in other epithelialized surfaces including the airways and the digestive tract. The *Lamc2* mutation results in systemic effects including failure to thrive and shortened lifespans. While we think it unlikely that body weight would *directly* affect tail tension values (and vice versa), it is reasonable to expect that body size/weight may covary with tail tension measurements. We have thus revised page 11 accordingly.

“Page 14, Line 230: The authors refer to a specific dystonin isoform (Dst-e) as a modifier candidate. More background should be provided on dystonin isoforms and why this one was specifically mentioned (presumably due to expression patterns?).”

Response: We have amended the text to clarify this concern.

“Figure 3A: It is interesting that several, perhaps the majority, of F2 mice exhibit a tail tension phenotype indicative of lower disease severity (higher tension) than either of the parental strains. What is the interpretation of this result?”

Response: The large spread in the 129XB6 F2 cohort is not unexpected given the number of F2 mice analyzed. The fact that the F2 mice were skewed towards higher tension values may be explained by the underlying genetics of that cross. The chr13 QTL appears to be weak for B6 vs 129X1, but chrs 11 and 13 are two QTL in which het phenotype matches the 129X1 phenotype (one allele dominant rather than typical semi-dominant). If chr13 was an important player and ¾ of F2s include its dominant phenotype because either BX or XX, that could explain an increase in resistant mice.

“Figure 3B: The extremely high LOD score for the chr1 QTL makes it somewhat difficult to appreciate the significance of other QTL. It may be beneficial to include a sub-panel with a y-axis similar to that of Fig. 4B-C or use a staggered y-axis.”

Response: A new panel 3C has been added to accomplish this.

Reviewer 2.

“Please provide the percentage of phenotypic variations (Tensions) explained by each QTL reported, which will help readers put into perspective the impact of each QTL on the outcome of EB.”

Response: Percentage variation has been added to newly created Table 1.

“The third effect plots in Fig 3C showed that mouse carrying allele BB has the highest tension, unlike the other three effect plots in the same figure. Could you please provide explanations.”

Response: New Table 1 summarizes this nicely and comments/explanations have been added to the Discussion.

“This study identifies potential DNA variants (SNPs/Indels) that may regulate EB outcomes, which will be useful for other researchers. Please provide a list of SNPs/Indels of interest as in Fig. 5J that were identified for the rest of QTLs.”

Response: A list for chr5/*Ppargc1a* candidate SNPs has been added as Fig 8F. Lists of chr11/*Itg4b* and chr13/*Dsp* candidates have been added as new Table 2. Additional comments have been added to the manuscript to clarify that the chr6 and chr10 candidate genes *Pparg* and *Igf1* (which are only listed because of their relationship to *Ppargc1a* – the chr6 and chr10 regions are large and contain many candidates though none clearly jump out as better than others) do not carry any SNPs with predicted allelic pattern FVB / MRL and B6 = 129X1 and good candidate functional consequences, such as missense or splice region variants. Therefore no chr6 or chr10 SNP candidates are offered in Table 2.
